# Early response to antibiotic treatment in European patients hospitalized with complicated skin and soft tissue infections: analysis of the REACH study

**DOI:** 10.1186/s12879-015-0822-2

**Published:** 2015-02-19

**Authors:** Javier Garau, Francesco Blasi, Jesús Medina, Kyle McBride, Helmut Ostermann, on behalf of the REACH study group

**Affiliations:** Department of Medicine, Hospital Universitari Mutua de Terrassa, Plaza Doctor Robert 5, 08221 Terrassa, Barcelona Spain; Department of Pathophysiology and Transplantation, University of Milan, IRCCS Fondazione Ospedale Maggiore, Policlinico Cà Granda Milano, Milan, Italy; Observational Research Centre, AstraZeneca, Parque Norte, Edificio Roble, Serrano Galvache 56, 28033 Madrid, Spain; Instat Services, Inc, 1 Wilson Street, Chatham, NJ 07928 USA; Department of Internal Medicine III, Haematology and Oncology, University Hospital Munich, Munich, Germany

**Keywords:** Complicated skin and soft tissue infections, Early response, Resource use, Epidemiology, Management, Treatment failure, Outcomes

## Abstract

**Background:**

The treatment of complicated skin and soft tissue infections (cSSTI) is challenging and many patients do not receive adequate first-line therapy. REACH (**RE**trospective Study to **A**ssess the **C**linical Management of Patients With Moderate-to-Severe cSSTI or Community-Acquired Pneumonia in the **H**ospital Setting) was a retrospective observational study of cSSTI patients in real-life settings in European hospitals. In this analysis, we review characteristics and outcomes of patients with an early response (≤72 hours) compared with those without an early response to treatment. We also compare the results according to two differing definitions of early response, one of which (Definition 1) requires resolution of fever within 72 hours, in line with previous US FDA guidelines.

**Methods:**

Patients were adults hospitalized with cSSTIs 2010–2011 and requiring treatment with intravenous antibiotics. Clinical management, clinical outcomes and healthcare resource use were assessed using a descriptive analysis approach.

**Results:**

The analysis set included 600 patients, of which 363 showed early response with Definition 1 and 417 with Definition 2. Initial treatment modification was frequent, and highest in patients without early response (48.1% with Definition 1). Patients without early response were more likely to have diabetes than those with early response (31.6% vs. 22.9%, respectively) and to suffer from more severe disease (e.g. skin necrosis: 14.8% and 7.7%, respectively), to be infected with difficult-to-treat microorganisms and to have recurrent infections. Furthermore, patients without early response had a higher rate of adverse clinical outcomes (e.g. septic shock) and higher use of healthcare resources. The results obtained with the two definitions for early response were largely similar.

**Conclusions:**

This study highlights the significance of early evaluation of patients in hospitals, in potentially preventing prolonged use of inappropriate or ineffective antibacterial therapy.

**Trial registration:**

NCT01293435.

**Electronic supplementary material:**

The online version of this article (doi:10.1186/s12879-015-0822-2) contains supplementary material, which is available to authorized users.

## Background

Complicated skin and soft tissue infections (cSSTIs) represent a heterogeneous range of diseases, from severe infections affecting otherwise healthy patients, to relatively minor infections affecting patients with several comorbidities [1]. cSSTIs are reported to be among the most common infections treated in the hospital setting [2], both in the UK, where they account for at least 10% of admissions to infection units [3], and in the USA, where hospital admissions for cSSTI increased by 29% from 2000–2004 [4].

Treatment of cSSTIs is typically empirical, and earlier studies have shown high rates of initial treatment failure in patients hospitalized with cSSTI [5,6]. Until recently, clinical trials evaluating antibacterial agents for treatment of cSSTI incorporated clinical cure as a primary endpoint. Clinical cure has traditionally been defined as total resolution of all signs and symptoms of the infection or improvement to such an extent that no further antimicrobial therapy is necessary [7,8]. In 2010, the US Food and Drug Administration (FDA) issued draft guidance recommending a new primary endpoint for industrial development of antimicrobials used for the treatment of acute bacterial skin and soft structure infection (ABSSSI) to be defined at 48–72 hours instead of the traditional test-of-cure [9]. This earlier time point could be more clinically relevant, as it would allow early identification of treatment success or failure and prevent prolonged use of inappropriate or ineffective antibacterial therapy, which is shown to be associated with adverse outcomes [6]. The FDA recommended endpoint includes co-primary outcomes of the resolution of fever and the cessation of the spread of the lesion after approximately 48–72 hours of antibacterial therapy [9].

The REACH study systematically collected real-life, current (2010–2011), pan-European data, on patients hospitalized for cSSTIs. This subanalysis aimed to evaluate the characteristics of patients with an early response to treatment (≤72 hours) compared with those without, according to two differing definitions of early response, and to identify any impact of an early response on clinical and economic outcomes.

## Methods

REACH (NCT01293435) was a retrospective, observational study of patients hospitalized with cSSTI and receiving intravenous (IV) antibiotic treatment. It enrolled 1,995 patients aged ≥18 years, from 129 sites in 10 participating countries across Europe (Belgium, France, Germany, Greece, Italy, the Netherlands, Portugal, Spain, Turkey and the UK). Further information on hospital sites is provided in Additional file 1: Table S1. Data detailing patient demographics, disease characteristics, microbiological diagnosis, disease course and outcomes, treatments before and during hospitalization and health resource consumption were collected via an electronic Case Report Form (eCRF). The study was performed according to Good Clinical Practice and the Declaration of Helsinki. All local ethics committees approved the study protocol. A list of ethics committees is provided in Additional file 2. Local legislation relating to written informed consent for non-interventional studies was followed in each country; in Germany and Portugal, where this information is mandatory, written informed consent was collected. Patients were required to have an infection affecting deeper soft tissue and/or requiring significant surgical intervention, an infection developing on a lower limb in subjects with diabetes mellitus or well-documented peripheral vascular disease, a major abscess, an infected ulcer, or deep and extensive cellulitis. Study design and patient inclusion and exclusion criteria are described in the primary publication for this study [10]. In short, patients were selected from the total number of patients admitted to hospital within that time frame with cSSTI, using an automatic randomization tool. The selected patients were then assessed for eligibility by conducting a first review of the medical charts. Patients who did not meet the predefined criteria of cSSTI (detailed in Additional file 1) or who did not require IV antibiotics were excluded. The rest were enrolled. Further inclusion and exclusion criteria are detailed in Additional file 1.

Initial treatment modification (ITM) was defined as a change from initial antibiotic treatment to a new antibiotic treatment due to insufficient response, adverse reaction, interaction with other drugs, non-suitability of the initial antibiotic based on the results of microbiological tests, changes in antibiotic therapy, or addition of further agents alone or in combination. No time limit was included in the definition. Cases of streamlining or de-escalation (defined as a change to narrower-spectrum antibiotics upon patient improvement or confirmed microbiological diagnosis) were not counted as ITM [10].

This subanalysis focuses on the characteristics, antibiotic treatments, clinical outcomes and use of healthcare resources of patients from the REACH study who achieved early response to treatment, compared with those who did not, as assessed by the responses to the following questions in the eCRF:

- Q1: Resolution of fever within the 72-hour period since initial antibiotic therapy;
- Q2: Documented indication of lesion improvement within the 72-hour period since initial antibiotic therapy;
- Q3: Cessation of spread of redness, oedema and/or induration of lesion within the 72-hour period since initial antibiotic therapy;
- Q4: Reduction in size of redness, oedema and/or induration within the 72-hour period since initial antibiotic therapy;
- Q5: Disappearance of local signs/symptoms present at admission within the 72-hour period since initial antibiotic therapy.

Early response was evaluated by two definitions; Definition 1 (D1; in line with the FDA draft guidance) [9] required resolution of fever and some indication of lesion improvement or stability within 72 hours of treatment initiation, i.e. a positive response to Q1 and Q2, Q3 or Q4. To address Q1, patients had to have fever at presentation. Definition 2 (D2) required evidence of lesion improvement or stability, or resolution of signs and symptoms within 72 hours, i.e. a positive response to Q2, Q3, Q4 or Q5, but did not include fever resolution as a requirement, as suggested by the Foundation for the National Institute of HealthBiomarkers Consortium [11].

Characteristics of patients showing early response to treatment (≤72 hours), defined by both D1 and D2, were compared with those of patients without early response, and corresponding outcomes and resource use measured. Only patients with available data in their medical records, sufficient to be classified according to each of these definitions, were included in this subanalysis.

### Statistical methods

This was a non-interventional study, using a descriptive analysis approach to assess clinical management, clinical outcomes and healthcare resource use.

## Results

### Patient population

**Figure 1:**
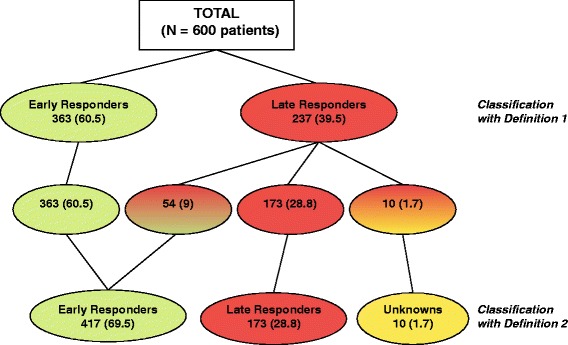
**Patient classification with Definition 1 and Definition 2.** Green denotes patients with an early response to treatment (≤72 hours), red denotes patients without an early response and yellow is used for unknowns. Mixed colours indicate those patients who had a different classification with the two definitions.

Of 1,995 patients enrolled in the REACH study, 1,513 (76%) had information detailing response to treatment recorded (Additional file 1: Table S2). The analysis set included 600 patients who had available data for assessment by D1 (Figure 1). A total of 363 (60.5%) of these patients were classified as early responders, while 237 (39.5%) were not. When D2 was used, an additional 54 (9%) patients were classified as early responders. This change in response outcome under the alternative definition was due to the fever resolution criterion in Q1 (47 patients) and to symptom resolution in Q5 (7 patients). Regardless of the consideration of fever resolution, 553/600 patients (92.2%) were included in the same classification. Therefore, including fever resolution restricted the number of patients who met the definition for early response, but the outcomes were largely unaffected.

Patient demographics, medical history and disease characteristics are shown in Table 1. The most common lesion types were cellulitis/fasciitis (approximately half of patients); abscess (one-fifth); and post-traumatic wound, post-surgical wound, diabetic leg ulcer or peripheral vascular disease ulcer (all around 5–10%). Approximately 20–25% had a recurrent skin infection and 8–10% a nosocomial infection. Demographics were similar between patients with and without an early response by both definitions, but patients without an early response were more likely to have diabetes than patients with (D1: 31.6% and 22.9%, respectively), and more severe disease (e.g. skin necrosis: 14.8% and 7.7%, respectively with D1).

**Table 1:** **Patient demographics, medical history and disease characteristics**

	**Definition 1**		**Definition 2**	
**Characteristic, n (%)**	**Early responders**	**Not early responders**	**Early responders**	**Not early responders**
	**n = 363**	**n = 237**	**n = 417**	**n = 173**
Age, years, mean (SD) [median]	58.5 (17.72) [58.0]	58.2 (17.17) [59.0]	58.9 (17.78) [58.0]	57.2 (17.08) [58.5]
<65 years	227 (62.5)	146 (61.6)	253 (60.7)	114 (65.9)
≥65 years	136 (37.5)	90 (38.0)	164 (39.3)	58 (33.5)
Sex, male	211 (58.1)	143 (60.3)	239 (57.3)	108 (62.4)
Relevant medical conditions affecting <10% of patients at hospitalization (initial visit*), n (%)				
Diabetes	83 (22.9)	75 (31.6)	103 (24.7)	50 (28.9)
Cancer/malignancy	62 (17.1)	29 (12.2)	69 (16.5)	19 (11.0)
Peripheral vascular disease	55 (15.2)	37 (15.6)	68 (16.3)	20 (11.6)
Congestive heart disease	47 (12.9)	30 (12.7)	58 (13.9)	18 (10.4)
Other relevant conditions^†^	100 (27.5 )	84 (35.4)	121 (29.0)	57 (32.9)
Unknown	1 (0.3)	1 (0.4)	1 (0.2)	1 (0.6)
Age of patients with comorbidities, years, mean (SD) [median]	62.2 (16.3) [62.0]	62.0 (15.1) [62.0]	62.8 (16.2) [63.5]	60.6 (15.2) [60.0]
Immunosuppressants/Immunomodulators in the 3 months prior to hospitalization, n (%)	35 (9.6)	17 (7.2)	40 (9.6)	11 (6.4)
Type of lesion^‡^				
Cellulitis/fasciitis	252 (69.4)	155 (65.4)	280 (67.1)	122 (70.5)
Abscess	77 (21.2)	45 (19.0)	86 (20.6)	34 (19.7)
Post-traumatic wound	40 (11.0)	18 (7.6)	48 (11.5)	10 (5.8)
Post-surgical wound	40 (11.0)	30 (12.7)	50 (12.0)	19 (11.0)
Diabetic leg ulcer	19 (5.2)	24 (10.1)	27 (6.5)	16 (9.2)
Peripheral vascular disease ulcer	14 (3.9)	20 (8.4)	19 (4.6)	14 (8.1)
Lesion extension >50 cm^2^	61 (16.8)	49 (20.7)	73 (17.5)	36 (20.8)
Lower extremities affected	227 (62.5)	180 (75.9)	265 (63.5)	134 (77.5)
Swelling/induration	239 (65.8)	173 (73.0)	273 (65.5)	132 (76.3)
Skin necrosis	28 (7.7)	35 (14.8)	38 (9.1)	24 (13.9)
Recurrent skin infection episode^§^	75 (20.7)	55 (23.2)	83 (19.9)	43 (24.9)
Nosocomial infection	30 (8.3)	22 (9.3)	35 (8.4)	17 (9.8)

*Visit to hospital for current infection or date of diagnosis of infection for patients already hospitalized.

^†^As defined by the investigator.

^‡^Patients could be classified with more than one type of cSSTI lesion.

^§^Patients hospitalized again due to same cSSTI.

SD = standard deviation.

Analysis of the patients evaluated by D1, compared with those who could not be evaluated, suggests that the evaluated patients were slightly younger, less likely to be female, and had fewer comorbidities, with a lower proportion having diabetic leg ulcers, peripheral vascular disease ulcers, fascia affected and skin necrosis. There was also a lower proportion of patients with recurrent skin infections in this group but no notable differences in microbiological diagnosis (Additional file 1: Table S3).

### Microbiological diagnosis

A microbiological diagnosis was available for around half of the patients in this subanalysis; patients without an early response were more likely to have a microbiological diagnosis with both definitions (Table 2). Patients with an early response were more likely to be infected with a Gram-positive microorganism compared with patients without (D1: 80.7% and 68.5%, respectively). Difficult-to-treat microorganisms and strict anaerobic bacteria were more frequently isolated from patients without an early response compared to those with. For methicillin-resistant *Staphylococcus aureus* (MRSA), this was only true when early response was assessed by D1 (13.1% and 9.9%, respectively). The numbers of patients with bacteraemia were low; however, a greater difference was seen between patients with and those without early response when assessed by D1 (D1: 5.5% and 9.3%, respectively; D2: 6.5% and 6.9%, respectively).

**Table 2:** **Microbiological diagnosis**

	**Definition 1**		**Definition 2**	
**Characteristic, n (%)**	**Early responders**	**Not-early responders**	**Early responders**	**Not-early responders**
	**n = 363**	**n = 237**	**n = 417**	**n = 173**
Investigations or diagnostic test				
Blood cultures	231 (63.6)	165 (69.6)	270 (64.7)	117 (67.6)
Superficial swab and culture	153 (42.1)	131 (55.3)	183 (43.9)	96 (55.5)
Needle aspiration	31 (8.5)	25 (10.5)	36 (8.6)	19 (11.0)
Surgical sample	38 (10.5)	37 (15.6)	48 (11.5)	25 (14.5)
Positive microbiological diagnosis	161 (44.4)	130 (54.9)	193 (46.3)	91 (52.6)
Gram-positive cocci*	130 (80.7)	89 (68.5)	156 (80.8)	56 (61.5)
Methicillin-sensitive *Staphylococcus aureus*	54 (33.5)	32 (24.6)	61 (31.6)	22 (24.2)
Methicillin-resistant *Staphylococcus aureus*	16 (9.9)	17 (13.1)	23 (11.9)	8 (8.8)
Coagulase-negative *Staphylococcus*	24 (14.9)	11 (8.5)	27 (14.0)	8 (8.8)
*Streptococcus pyogenes* (group A *β*-haemolytic streptococci)	10 (6.2)	6 (4.6)	13 (6.7)	2 (2.2)
*Streptococcus agalactiae* (group B *β*-haemolytic streptococci)	4 (2.5)	8 (6.2)	7 (3.6)	5 (5.5)
Other *β*-haemolytic streptococci^†^	13 (8.1)	7 (5.4)	14 (7.3)	4 (4.4)
*Streptococcus pneumoniae*	1 (0.6)	0 (0)	1 (0.5)	0 (0)
*Enterococcus faecalis*	11 (6.8)	6 (4.6)	12 (6.2)	4 (4.4)
*Enterococcus faecium*	4 (2.5)	3 (2.3)	5 (2.6)	2 (2.2)
Other Gram-positive bacteria^‡^	4 (2.5)	5 (3.8)	4 (2.1)	5 (5.5)
*Enterobacteriaceae* ^§^, other Gram-negative bacteria^¶^, other strict anaerobic bacteria^**^	53 (32.9)	46 (35.4)	60 (31.1)	37 (40.7)
Non-fermenting Gram-negative bacilli^††^	16 (9.9)	15 (11.5)	20 (10.4)	11 (12.1)
Polymicrobial infections	47 (29.2)	29 (22.3)	53 (27.5)	19 (20.9)
Yeasts	2 (1.2)	3 (2.3)	2 (1.0)	3 (3.3)
Other microorganisms	4 (2.5)	1 (0.8)	4 (2.1)	1 (1.1)
Bacteraemia	20 (5.5)	22 (9.3)	27 (6.5)	12 (6.9)

*Includes subgroups below and *Staphylococcus warnerii, Staphylococcus lugdugensis, Staphylococcus haemolyticus, Staphylococcus epidermidis, Staphylococcus* spp. non-*aureus, Streptococcus mitis, Streptococcus constellatus,* viridans *Streptococcus,* Group G streptococci, *Streptococcus mitis, Enterococcus* spp., unspecified Gram-positive cocci.

^†^Includes *S. dysgalactiae*, Group C streptococci, microaerophilic streptococci, *S. mileri*, *S. intermedius*, *S. anginosus*, *S. bovis*.

^‡^Includes *Bacillus anthracis*, *Corynebacterium* spp*.*, diphtheroids, *Proprionibacterium* spp., *Lactobacillus* spp., *Clostridium* spp., Gram-positive bacilli non-specified.

^§^Includes *Proteus mirabilis, Escherichia coli, Klebsiella* spp, *Enterobacter* spp, *Citrobacter* spp, *Serratia marcescens, Providencia stuartii, Morganella morganii, Pantoea* spp.).

^¶^Include*s Neisseria* spp, *Aeromonas hydrophila*, *Pasteurella multocida.*

^**^Includes *Gemella morbillorum, Bacteroides fragilis, Peptostreptococcus* spp., *Prevotella melaninogenica, Porphyromonas* spp.

^††^Includes *Pseudomonas* spp.*, Acinetobacter* spp*., Stentrophomonas maltophilia, Shweanella putrefacians.*

### Treatment characteristics

Most patients received empiric first-line treatment (D1: 90.1% and 73.4% with and without early response, respectively – Table 3) and the majority of treatments were initiated on the first day of hospitalization. Around half of patients received only one course of antibiotic therapy (Additional file 1: Table S4). Treatment characteristics were similar with both definitions.

**Table 3:** **Most frequent antibiotics used as initial therapy (monotherapy and combinations)**

	**Definition 1**		**Definition 2**	
**Antibiotic, n (%)**	**Early responders**	**Not-early responders**	**Early responders**	**Not-early responders**
	**n = 363**	**n = 237**	**n = 417**	**n = 173**
Empiric treatment	327 (90.1)	174 (73.4)	368 (88.2)	126 (72.8)
Amoxicillin–clavulanate	66 (18.2)	29 (12.2)	70 (16.8)	24 (13.9)
Piperacillin–tazobactam	20 (5.5)	20 (8.4)	24 (5.8)	16 (9.2)
Ampicillin–sulbactam	25 (6.9)	47 (19.8)	30 (7.2)	42 (24.3)
Cloxacillin	16 (4.4)	4 (1.7)	18 (4.3)	1 (0.6)
Clindamycin	14 (3.9)	3 (1.3)	15 (3.6)	0 (0)
Penicillins or combinations + fluoroquinolone	11 (3.0)	6 (2.5)	12 (2.9)	5 (2.9)
Cefuroxime	11 (3.0)	5 (2.1)	14 (3.4)	2 (1.2)
Fluoroquinolone	6 (1.7)	4 (1.7)	6 (1.4)	4 (2.3)
Daptomycin	11 (3.0)	4 (1.7)	11 (2.6)	4 (2.3)
Carbapenem	9 (2.5)	9 (3.8)	10 (2.4)	7 (4.0)

There were few notable differences in antibiotic therapy used, except that more patients with an early response were treated with amoxicillin–clavulanate as initial therapy, compared with those without, and more patients without an early response were treated initially with ampicillin–sulbactam or piperacillin–tazobactam than those with early response (Table 3).

### Clinical outcomes and resource use

Clinical outcomes and resource use are detailed in Table 4. ITM was high in all groups, but was higher in patients without an early response, with almost half of these requiring ITM (D1: 34.2% and 48.1% with and without early response, respectively). Overall treatment duration, incidence of surgery after diagnosis, reinfection or recurrence, complications (e.g. septic shock and acute renal failure) and mortality were also all higher in patients without an early response.

**Table 4:** **Clinical outcomes and resource use**

	**Definition 1**		**Definition 2**	
**Clinical outcomes and resource use**	**Early responders**	**Not-early responders**	**Early responders**	**Not-early responders**
	**n = 363**	**n = 237**	**n = 417**	**n = 173**
Initial treatment modification, n (%)	124 (34.2)	114 (48.1)	148 (35.5)	87 (50.3)
Overall treatment duration, days, mean (SD) [median]	10.8 (8.7) [9.0]	17.6 (15.3) [13.0]	11.3 (10.2) [9.0]	18.4 (14.7) [14.0]
Length of hospital stay, days, mean (SD) [median]	14.3 (16.7) [9.0]	22.8 (24.5) [16.0]	14.8 (17.3) [10.0]	24.3 (25.7) [18.0]
Surgery after diagnosis, n (%)	98 (27.0)	91 (38.4)	121 (29.0)	63 (36.4)
Reinfection or recurrence, n (%)	29 (8.0)	24 (10.1)	34 (8.2)	16 (9.2)
Admitted to ICU, n (%)	15 (4.1)	38 (16.0)	27 (6.5)	26 (15.0)
Time in ICU, days, mean (SD) [median]	4.4 (4.6) [3.5]	10.1 (15.3) [5.0]	6.6 (9.7) [4.0]	10.4 (16.4) [5.0]
Acute renal failure, n (%)	2 (0.6)	14 (5.9)	4 (1.0)	12 (6.9)
Length of acute renal failure, days, mean (SD) [median]	3.5 (2.1) [3.5]	20.5 (54.2) [4.0]	3.0 (1.7) [2.0]	22.0 (56.3) [4.5]
Blood pressure support, n (%)				
Fluid resuscitation	20 (5.5)	36 (15.2)	27 (6.5)	28 (16.2)
Vasopressors	3 (0.8)	22 (9.3)	6 (1.4)	19 (11.0)
Invasive procedures	0	0	0	0
Septic shock, n (%)	3 (0.8)	15 (6.3)	6 (1.4)	12 (6.9)
Isolation required, n (%)	7 (1.9)	18 (7.6)	13 (3.1)	11 (6.4)
Parenteral nutrition, n (%)	9 (2.5)	16 (6.8)	11 (2.6)	13 (7.5)
Length of parenteral nutrition, days, mean (SD) [median]	10.7 (10.2) [6.0]	38.4 (71.3) [12.0]	11.6 (11.3) [6.0]	43.9 (77.8) [13.0]
Home-based care after discharge, n (%)	45 (12.4)	31 (13.1)	54 (12.9)	20 (11.6)
Mortality, n (%)	5 (1.4)	9 (3.8)	6 (1.4)	8 (4.6)

ICU = Intensive care unit.

Economic outcomes and use of healthcare resources were associated with Day 3 clinical response, regardless of the definition used. Patients without early response to treatment had a higher rate of admission to the intensive care unit compared to patients with early response (D1: 16.0% and 4.1%, respectively) and a median 7 or 8 days’ longer hospital stay. Findings were similar with both definitions (Table 4).

## Discussion

Many patients with cSSTI do not receive adequate first-line treatment [5,6]. Our subanalysis found that early response to treatment is associated with better clinical outcomes and lower use of hospital resources. These results are consistent with previous findings. A retrospective cohort analysis of ABSSSI patients in New Jersey showed that the cost of care for patients without early response to antibiotic treatment was >1.5-fold higher than for patients with early response (*p* < 0.0001) and patients who did not respond within 72 hours had an additional 3.7 days of treatment (*p* < 0.0001) [12].

In an earlier retrospective study, inappropriate initial therapy was associated with cSSTI caused by mixed pathogens or MRSA, or by pathogens other than *S. aureus* or streptococci [13]; however, another study found that the incidence of MRSA did not differ between groups [14]. In our study, patients without an early response were more likely to be infected with Gram-negative bacteria and anaerobes, whereas Gram-positive bacteria were more frequently isolated from early responders.

The rate of ITM in our subanalysis was higher than previously observed, with almost half of the patients without an early response requiring initial treatment to be changed. Importantly, ITM was highest in patients who did not respond to treatment within 72 hours. The majority of patients were treated empirically. Two large multi-centre retrospective studies in the US have shown that in patients hospitalized with cSSTI, initial treatment failure is frequent (19.4–22.8%) [5,6]. Berger *et al*. also showed that patients with initial treatment failure have 4- to 12-fold higher mortality rates, spend 4.1–7.3 additional days in hospital and incur $11,995–$23,655 additional inpatient costs [5]. Data from a smaller, single-centre, retrospective study suggest inappropriate treatment is associated with increased use of healthcare resources (e.g. longer hospitalization) but not with clinical outcomes such as mortality [14]; however, these associations may vary depending on the type of cSSTI evaluated [13].

The observation that later response to treatment is associated with worse clinical outcomes and higher use of healthcare resources highlights the need to identify patients less likely to respond early to treatment, in order to improve care and limit complications. One of the objectives of this subanalysis was to compare the results obtained with two definitions of early response, one of which (D1) required resolution of fever within 72 hours [9]. The other definition (D2) did not require fever resolution, based on the fact that fever is not on the causal pathway of the disease and that a requirement for fever presentation at baseline may exclude certain populations, such as older patients, from participating in clinical trials [11]. The results were largely similar, but overall, D1 appeared to provide more specific differentiation between patients with and without early response, although the reason for this difference is not apparent. Patients evaluated in our subanalysis were younger and had fewer comorbidities and less severe disease compared with those who could not be evaluated. This highlights a limitation of our subanalysis, as the evaluated population may not be representative of all patients in the REACH study. These observations might reflect the requirement for fever at presentation as an inclusion criterion for this subanalysis, which may exclude certain populations from being assessed [11]. In this study, information on fever resolution was unavailable in the medical records of 39% of patients (slightly higher than the rate of unavailable information for Q2, Q3, Q4 and Q5).

Another objective of the study was real-life observation of cSSTI in Europe, which by definition results in the inclusion of a heterogeneous patient population with associated limitations, but has enabled us to capture a picture of current practice which has highlighted real concerns. There are no recent European treatment guidelines for cSSTI. Treatment is often empiric and selection of first-line treatment is highly variable. This may be driven by a broad range of potential pathogens, the need to treat often without a confirmed microbiological diagnosis, and a large generic pool of treatment options [6]. The findings of REACH confirm this, with 54 different initial antibiotic regimens (monotherapy or combination) used and the majority of patients treated empirically [10]. In addition, although all patients in this study underwent a microbiological test, a large proportion of specimens were superficial swabs and therefore the culture results may largely reflect colonization. These are concerning findings which suggest an improvement in antibiotic stewardship is needed urgently, along with early identification of patients at increased risk, to optimize selection of the most suitable antibiotic treatment.

In medical practice, Day 3 clinical endpoints can have strong therapeutic relevance. Early indications of treatment failure can guide antimicrobial treatment modification within 72 hours, thus avoiding prolonged use of inappropriate antimicrobials and/or help to recognize the need for surgery. Evaluation at Day 3 of the clinical evolution (course) and availability of the results of initial cultures can aid decisions to de-escalate treatment to a narrower-spectrum agent or to switch from IV to oral therapy, evaluate the need for surgery, and subsequently discharge a patient based on clinical improvement. A number of recent studies have incorporated the FDA-recommended endpoint of response to antimicrobial treatment within 72 hours. In their retrospective analysis of data from the CANVAS 1 and 2 clinical trials, Friedland *et al*. showed that ceftaroline fosamil treatment for ABSSSI led to a higher clinical response compared with vancomycin plus aztreonam at this early endpoint [15]. The CANVAS trials employed stricter inclusion criteria for skin infections than the REACH study, excluding diabetic foot ulcers and necrotising infections. Recently, a clinical trial reported the non-inferiority of tedizolid phosphate to linezolid for ABSSSI at 48–72 hours [16]. In addition, a retrospective analysis of vancomycin for treatment of MRSA bloodstream infections found that lack of response at Day 3 was the strongest predictor of end-of-treatment failure [17]. These findings suggest that Day 3 endpoints are useful efficacy endpoints in the design of clinical trials for antimicrobial agents for treatment of cSSTI.

## Conclusion

This retrospective analysis of observational data from patients hospitalized with cSSTI highlights the real-life significance of an early response to treatment in terms of better clinical outcomes and reduced use of healthcare resources.

## Additional files

Additional file 1:
**Inclusion and exclusion criteria. Table S1.** Participating hospital sites. **Table S2.** Patients who had information detailing their response to treatment recorded, by country. **Table S3.** Demographics and disease characteristics of patients who were evaluated by Definition 1 and those who could not be evaluated. **Table S4.** Treatment characteristics. Additional file 2:
**List of ethics committees.**
