# Supplementary material for: Early response to antibiotic treatment in European patients hospitalized with complicated skin and soft tissue infections: analysis of the REACH study
Source: BMC Infect Dis. 2015 Feb 19;15:78. doi: 10.1186/s12879-015-0822-2 (PMC4352248; doi:10.1186/s12879-015-0822-2)
Supplement: Additional file 1: — Inclusion and exclusion criteria. Table S1. Participating hospital sites. Table S2. Patients who had information detailing their response to treatment recorded, by country. Table S3. Demographics and disease characteristics of patients who were evaluated by Definition 1 and those who could not be evaluated. Table S4. Treatment characteristics. [file 12879_2015_822_MOESM1_ESM.docx]

**Additional File 1.doc**

**Inclusion and exclusion criteria**

Patients were adults ≥18 years of age, requiring initial hospitalization and receiving treatment with intravenous antimicrobials. Patients were required to have an infection affecting deeper soft tissue and/or requiring significant surgical intervention, infection developing on a lower limb in subjects with diabetes mellitus or well-documented peripheral vascular disease, a major abscess, infected ulcer or deep and extensive cellulitis. In addition, the presence of at least two local signs of cSSTI (purulent or seropurulent drainage/discharge, erythema, fluctuance, heat/localised warmth, pain/tenderness to palpation, swelling/induration) and at least one systemic sign (temperature of >38°C, white blood cell count of >10,000/mm^3^ or >10% immature neutrophils) was required.

Patients already participating in a clinical trial or any other interventional study were excluded. Patients with uncomplicated SSTIs, such as simple abscesses, impetiginous lesions, superficial cellulitis, furunculosis, carbunculosis or folliculitis were also excluded, as were patients with cSSTI with a high cure rate after surgical incision alone or after aggressive local skin care.

**Table S1 Participating hospital sites**

|  | **Early responders** | **Not-early responders** |
| --- | --- | --- |
| Number of hospital sites participating | 30 | 10 |
| Type of hospital |  |  |
| University Hospital | 17 (56.7) | 6 (60.0) |
| General Hospital | 9 (30.0) | 1 (10.0) |
| Regional Hospital | 4 (13.3) | 3 (30.0) |
| Type of funding |  |  |
| Public | 29 (96.7) | 9 (90.0) |
| Private | 1 (3.3) | 0 (0.0) |
| Unknown | 0 (0.0) | 1 (10.0) |
| Hospital size |  |  |
| Less than 150 beds | 2 (6.7) | 0 (0.0) |
| 151 to 300 beds | 3 (10.0) | 1 (10.0) |
| 301 to 400 beds | 0 (0.0) | 1 (10.0) |
| 401 to 500 beds | 2 (6.7) | 1 (10.0) |
| 501 to 600 beds | 3 (10.0) | 0 (0.0) |
| More than 600 beds | 20 (66.7) | 7 (70.0) |
| Type of specialist/department |  |  |
| Internist | 1 (3.3) | 2 (20.0) |
| Pneumologist | 2 (6.7) | 0 (0.0) |
| Infectious diseases specialist | 23 (76.7) | 4 (40.0) |
| Intensive Care Unit (ICU) specialist | 2 (6.7) | 1 (10.0) |
| Surgeon | 0 (0.0) | 2 (20.0) |
| Diabetologist | 0 (0.0) | 1 (10.0) |
| Other | 2 (6.7) | 0 (0.0) |

**Table S2 Patients who had information detailing their response to treatment recorded, by country**

| **Country** | **Number of patients (N=1,513)** |
| --- | --- |
| Italy | 299 |
| Spain | 298 |
| Turkey | 290 |
| Belgium | 187 |
| France | 125 |
| Greece | 99 |
| The Netherlands | 74 |
| United Kingdom | 69 |
| Germany | 39 |
| Portugal | 33 |

**Table S3 Demographics and disease characteristics of patients who were evaluated by Definition 1 and those who could not be evaluated**

| **Characteristic, n (%)** | **Definition 1** | | **Not evaluable by Definition 1**  **n=913** |
| --- | --- | --- | --- |
|  | **Early responders**  **n=363** | **Not-early responders**  **n=237** |  |
| Age, years, mean (SD) [median] | 58.5 (17.72) [58.0] | 58.2 (17.17) [59.0] | 60.8 (17.5) [63.0] |
| <65 years  ≥65 years | 227 (62.5) 136 (37.5) | 146 (61.6) 90 (38.0) | 486 (53.2)  427 (46.8) |
| Sex, male | 211 (58.1) | 143 (60.3) | 519 (56.8) |
| Any relevant condition | 259 (71.3) | 177 (74.7) | 740 (81.1) |
| Type of lesion*  Diabetic leg ulcer  Peripheral vascular disease ulcer  Fascia affected  Skin necrosis | 19 (5.2)  14 (3.9)  41 (11.3)  28 (7.7) | 24 (10.1)  20 (8.4)  61 (25.7)  35 (14.8) | 126 (13.8)  122 (13.4)  240 (26.3)  203 (22.2) |
| Recurrent skin infection episode^†^ | 75 (20.7) | 55 (23.2) | 263 (28.8) |
| Unknown  Positive microbiological diagnosis  Gram-positive cocci^‡^  MSSA  MRSA  *Enterobacteriaceae*^§^, other Gram negative bacteria^¶^, other strict anaerobic bacteria^**^  Non-fermenting gram negative bacilli^††^  Polymicrobial infections  Bacteremia | 202 (55.6)  161 (44.4)  130 (80.7)  54 (33.5)  16 (9.9)  53 (32.9)  16 (9.9)  47 (29.2)  20 (5.5) | 107 (45.1)  130 (54.9)  89 (68.5)  32 (24.6)  17 (13.1)  46 (35.4)  15 (11.5)  29 (22.3)  22 (9.3) | 433 (47.4)  480 (52.6)  326 (67.9)  125 (26.0)  49 (10.2)  184 (38.3)  60 (12.5)  156 (32.5)  52 (5.7) |

*Patients could be classified with more than one type of cSSTI lesion.

^†^Patients hospitalized again due to same cSSTI.

^‡^Includes subgroups below and *Staphylococcus warnerii, Staphylococcus lugdugensis, Staphylococcus haemolyticus, Staphylococcus epidermidis, Staphylococcus* spp. non-*aureus, Streptococcus mitis, Streptococcus constellatus,* viridans *Streptococcus,* Group G streptococci, *Streptococcus mitis, Enterococcus* spp., unspecified Gram-positive cocci.

^§^Includes *Proteus mirabilis, Escherichia coli, Klebsiella* spp, *Enterobacter* spp, *Citrobacter* spp, *Serratia marcescens, Providencia stuartii, Morganella morganii, Pantoea* spp.).

^¶^Include*s Neisseria* spp, *Aeromonas hydrophila*, *Pasteurella multocida.*

^**^Includes *Gemella morbillorum, Bacteroides fragilis, Peptostreptococcus* spp., *Prevotella melaninogenica, Porphyromonas* spp.

^††^Includes *Pseudomonas* spp.*, Acinetobacter* spp*., Stentrophomonas maltophilia, Shweanella putrefacians.*

SD = standard deviation.

**Table S4 Treatment characteristics**

|  | **Definition 1** | | **Definition 2** | |
| --- | --- | --- | --- | --- |
| **Characteristic, n (%)** | **Early responders**  **n=363** | **Not-early responders**  **n=237** | **Early responders**  **n=417** | **Not-early responders**  **n=173** |
| Time from hospitalization to first antibiotic , days, mean, (SD) [median] | 1.3 (6.02) [0] | 1.6 (6.53) [0] | 1.3 (5.86) [0] | 1.6 (6.76) [0] |
| Day of administration of 1^st^ antibiotic therapy  Before hospitalization  1^st^ day of hospitalization  2^nd^ day of hospitalization  3^rd^ day of hospitalization | 0  295 (81.3)  38 (10.5)  5 (1.4) | 1 (0.4)  186 (78.5)  21 (8.9)  7 (3.0) | 0  337 (80.8)  44 (10.6)  7 (1.7) | 0  136 (78.6)  15 (8.7)  5 (2.9) |
| Number of antibiotic therapy courses, n (%)  1  2  3  >3 | 183 (50.4)  135 (37.2)  26 (7.2)  19 (5.2) | 97 (40.9)  81 (34.2)  30 (12.7)  28 (11.8) | 205 (49.2)  156 (37.4)  31 (7.4)  25 (6.0) | 72 (41.6)  54 (31.2)  24 (13.9) 22 (12.7) |
